# Comparing Sediment Bacterial Communities of Volcanic Lakes and Surrounding Rivers in Inner Mongolia Autonomous Region, Northeastern China

**DOI:** 10.3390/microorganisms12071435

**Published:** 2024-07-15

**Authors:** Jianying Chao, Jian Li, Jing Gao, Chengrong Bai, Xiangming Tang, Keqiang Shao

**Affiliations:** 1 Nanjing Institute of Environmental Sciences, Ministry of Ecology and Environment, Nanjing 210042, China; cjy@nies.org (J.C.); vickylijian@foxmail.com (J.L.); gj113759@163.com (J.G.); 2 Shandong Key Laboratory of Eco-Environmental Science for Yellow River Delta, Shandong University of Aeronautics, Binzhou 256600, China; crbai@sdua.edu.cn; 3 State Key Laboratory of Lake Science and Environment, Nanjing Institute of Geography and Limnology, Chinese Academy of Sciences, Nanjing 210008, China; xmtang@niglas.ac.cn

**Keywords:** UNESCO Global Geopark, volcanic lake, river, sediment bacteria, network analysis

## Abstract

Volcanic lakes originate from a volcanic crater or caldera, and were a crucial component of aquatic ecosystems. Sediment bacteria play an important role in the nutrient cycling of aquatic ecosystems; however, their patterns distribution in volcanic lakes and the surrounding river habitats are unknown. In this study, we compare the sediment bacterial communities and their co-occurrence networks between these two habitats in the Inner Mongolia Autonomous Region, Northeastern China (the Arxan UNESCO Global Geopark), using 16S rRNA gene amplicon sequencing. The results revealed that there were significant variations in the physicochemical parameters of the sediment between these two habitats. The bacterial α-diversity, β-diversity, and community composition of the sediment also significantly differed between these two habitats. Network analysis showed that the co-occurrence patterns and keystone taxa in the sediment differed between these two habitats. The sediment bacterial communities in the river habitats were more stable than those in the lake habitats in the face of environmental change. Canonical correspondence analysis demonstrated that both physical (pH and MC) and nutrition-related factors (TN, TP, LOI, and TOC) were the most important environmental factors shaping the variations of bacterial community composition (BCC) in the sediment between these two habitats. This work could greatly improve our understanding of the sediment BCC of the sediment from aquatic ecosystems in the UNESCO Global Geopark.

## 1. Introduction

Lakes, as an essential component of the aquatic ecosystem, are widely spread around the world and provide a range of ecological functions [1]. Meanwhile, lakes have also been identified as early indicators of regional and global environmental changes caused by both climate change and human activities [2]. Sediment bacteria play important ecological and biogeochemical roles in the nutrient cycling of lake ecosystems [3,4]. They are believed to be sentinels that are sensitive to environmental changes and can serve as useful indicators of water pollution, as well as potential biomarkers for monitoring environmental stressors [5]. Therefore, studying the bacterial communities in sediments is an important step for better understanding the metabolic processes in lake ecosystems [6].

Volcanic lakes are distinguished significantly from other natural lakes by their long water renewal periods and a hypolimnetic zone that is larger than the epilimnetic zone, with a pronounced susceptibility to summer-autumn hypolimnetic anoxia and, in rare cases, meromixis. These lakes have recently been subjected to a variety of water quality deteriorations as a result of the urbanization of their watershed region, water capture for agricultural uses, and the increased recreational usage of the lakes. Until now, studies on these volcanic lakes have mostly focused on the age of the gasses at the time of the disaster, the reverse of the CO_2_ supersaturated hypolimnion [7,8], the composition and concentrations of the physicochemical components [9], the escape chronology of gasses, the existence of isotopes and rare gasses [10], the degassing process [11], and the diversity and structure of prokaryotic communities [12]. However, in the volcanic lakes and surrounding rivers, the data on bacterial communities of the sediment are still little regarded.

Arxan (119°28′~121°23′ E, 46°39′~47°39′ N), a mountain city located in the southwest foothills of China’s Greater Khingan Mountains, attracts a large number of tourists each year. It is situated in the central part of the Greater Khingan Mountains [approximately 1400 km from north to south and an elevation of 1100 to 1400 m]. Arxan city has a total land area of 7409 km^2^, is characterized by a sub-humid cold temperate climate, and is located in a transitional zone between the eastern monsoon and arid region. Between 1953 and 2019, the annual average temperature at the Arxan meteorological station was −2.6 °C, with an annual average precipitation of 452 mm [13]. Arxan has numerous separated crater lakes and interconnecting lava barrier lakes [13]. However, there is currently little information about the BCC inhabiting the sediments of volcanic lakes and the surrounding rivers in this mountain catchment.

Therefore, the present study is the first to employ Illumina MiSeq sequencing of 16Sr RNA for the sediment bacterial communities of volcanic lakes and the surrounding river habitats in the Inner Mongolia Autonomous Region, Northeastern China. The aims of this study were to (i) compare the diversity and composition of the bacterial community in the sediment between these two habitats; (ii) characterize the architecture of both bacterial co-occurrence networks; and (iii) determine which major environmental factors control the variations of the sediment BCC between these two habitats.

## 2. Materials and Methods

### 2.1. Study Area and Sampling Sites

**Figure 1:**
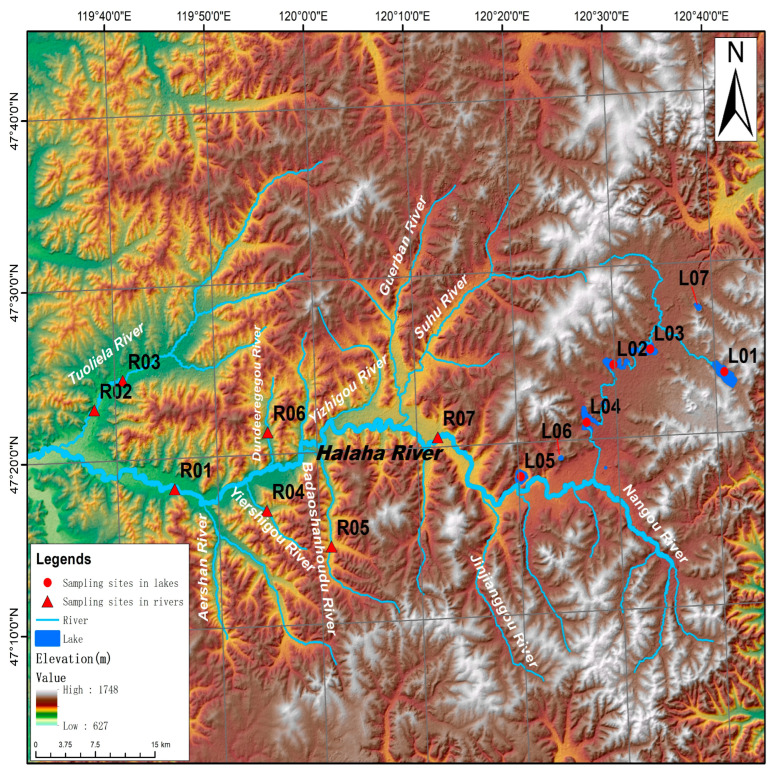
Map of the Arxan UNESCO Global Geopark, China, showing the location of 14 sampling sites.

“Arxan” means “Holy Hot Spring” in Mongolian. The Arxan UNESCO Global Geopark, situated in the Greater Khingan Mountains, is China’s biggest volcanic hot spring geopark, covering roughly 3653 km^2^. This geopark features volcanic remnants, hot spring landforms, alpine lakes, and meandering rivers, and the forest coverage rate is higher than 80%. In this study, we focused on volcanic lakes and the surrounding river habitats within the Arxan UNESCO Global Geopark, and worked at 14 sampling sites as follows: L01, L02, L03, L04, L05, L06, L07 in the lake habitats, and R01, R02, R03, R04, R05, R06, R07 in the river habitats (Figure 1). The longitude and latitude of each sampling site are provided in Table S1. 

### 2.2. Sampling and Geochemistry Measurements

Three surface sediments (0–2 cm) were collected with a peterson sediment sampler from each sampling site on 16 June 2022. The sediment samples were immediately placed into sterile plastic containers and dried in the laboratory using a freeze dryer (LABCONCO, Kansas, MO, USA). Some samples were stored at −80 °C until DNA extraction, and the remaining was used for chemical analysis. The pH was measured in situ by specific electrodes (REX, PHB-5, INESA, Shanghai, China). Standard methods were employed to measure the total nitrogen (TN), total phosphorus (TP), loss on ignition (LOI), moisture content (MC), and total organic carbon (TOC).

### 2.3. DNA Extraction and Illumina Miseq Sequencing

Following the manufacturer’s instructions, sediment DNA was extracted from 0.5 g solid sample (dry weight) using the MoBio PowerSoil™ DNA isolation kit (MoBio, Carlsbad, CA, USA). The 16S rRNA gene was amplified by a polymerase chain reaction (PCR) using the primer set 341F (5′-CCTACGGGNGGCWGCAG-3′) and 806R (5′-GGACTACHVGGGTATCTAAT-3′) targeting the hypervariable bacterial V3–V4 region. The amplified 16S rRNA genes for each sample were pooled in triplicate after purification. After determining the amplicon content, equimolar amounts of barcoded amplicons for each sample were sequenced on an Illumina MiSeq PE300 platform by Shanghai Personal Biotechnology (Co., Ltd.), Shanghai, China.

### 2.4. Bioinformatics Analysis and Network Construction and Analysis

Bioinformatic analysis, such as trimming primer sequences, eliminating chimeras, the clustering of operational taxonomic units (OTUs), filtering low abundance OTUs (<10 reads), and taxonomic assignment based on SILVA database 138, were conducted in the CLC Microbial Genomics Module [14,15].

The species co-occurrence patterns of both lake and river habitats were constructed using the network theory to obtain a better understanding of the relevance of interspecies interaction to community assembly [16,17,18]. Only OTUs with a relative abundance greater than 0.03% in lake/river samples were chosen for further analysis to improve the reliability of the network analysis and simplify the dataset. Subsequently, only OTUs detected in more than 50% of the samples were considered for network construction. The network analyses were performed by the Pearson correlation coefficient and a random matrix theory (RMT) modeling for threshold identification, based on the online Molecular Ecological Network Analysis (MENA) pipeline “http://ieg4.rccc.ou.edu/mena/ (accessed on 12 December 2023)” [17].

The “psych” package was used to compute the significance matrix (*p*-values) and the correlation matrix (R-values). Spearman’s rank correlations were employed for these calculations. Focusing on species–species interactions, only spearman correlation coefficients with an absolute value larger than 0.80 and strong correlations (*p* ≤ 0.01) were chosen for the subsequent network analysis. The “igraph” package in R was used to quantify network topological attributes, such as the average degree (avgK), average clustering coefficient (AvgCC), average degree (AD) and density (GD) [19]. A robustness analysis was performed on all networks to determine the network stability. Additionally, the co-occurrence networks were visualized by the interactive platform Gephi (v0.9.2).

### 2.5. Statistical Analysis

The bacterial α-diversity was calculated after normalizing the sequencing depth based on the smallest sequencing effort using Mothur software (version 1.31.2, http://www.mothur.org/ (accessed on 12 December 2023)). The bacterial β-diversity between the lake and river sediment samples were assessed by weighted Unifrac results in QIIME 2. Statistical analyses and visualizations were performed using R (v. 4.0.4) (https://www.r-project.org (accessed on 12 December 2023)) within the RStudio platform (v. 1.4.1717). The non-parametric Kruskal–Wallis rank test was used to compare the differences in bacterial α-diversity indices between these two habitats. Cluster analysis and metric multidimensional scaling (MMDS) based on the Bray–Curtis distance were performed using PRIMER v7 to visualize the β-diversity (compositional variation) between these two habitats. Differences in bacterial community structures between these two habitats were evaluated using permutational multivariate analysis of variance (PERMANOVA) based on Bray–Curtis dissimilarity with 999 permutations using the R package ‘vegan’. Redundancy analysis (RDA) was performed to explore the correlations between the environmental variables and the sediment BCC using the vegan package in R [20]. The OTUs data were Hellinger-transformed for all downstream analysis to reduce the influence of highly abundant OTUs [21]. Environmental variables were normalized to the multiple measurement scales. Then, a forward selection approach was performed using a Monte Carlo test with 999 permutations. Only variables that explained a significant (*p* < 0.05) increased fraction of total variation were considered in the subsequent selection. 

### 2.6. Nucleotide Sequence Accession Number

The sequencing data have been deposited at the Sequence Read Archive database of the National Center for Biotechnology Information (NCBI) under accession number PRJNA1032410.

## 3. Results 

### 3.1. Environmental Characterization

**Figure 2:**
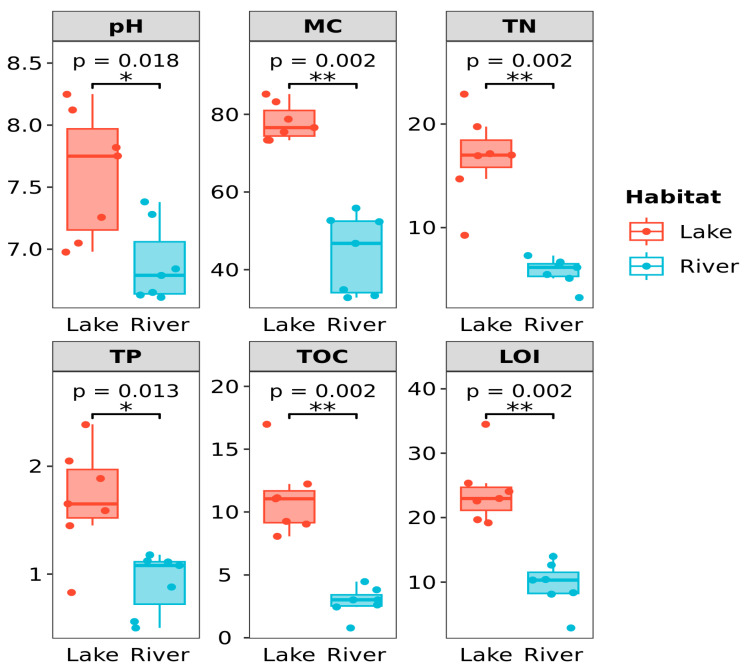
Comparisons of major physicochemical properties of the sediment between the lake and river habitats. *, *p* < 0.05; **, *p* < 0.01.

Highly significant differences were observed in five environmental parameters (pH, TN, LOI, MC, and TOC) of the sediment between these two habitats (*p* < 0.01, Figure 2). The pH of the sediment in the lake and river habitats was 6.88 ± 0.32 and 6.20 ± 0.15, respectively. The concentrations of TN ranged from 9.26 g/kg (L01) to 22.89 g/kg (L05) in the lake habitats, while the river habitats varied from 3.24 g/kg (R03) to 7.30 g/kg (R02). The MC value ranged from 73.33% (L01) to 85.21% (L02) in the lake habitats, while the river habitats varied from 32.81% (R04) to 55.84% (R01). The LOI value ranged from 19.18 (L04) to 34.49 (L05) in the lake habitats, while the river habitats varied from 2.88 (R04) to 13.99 (R01). The concentrations of TOC in these two habitats ranged between 8.06 (L04) and 16.98 (L05) and between 0.78 (R04) and 4.47 (R02), respectively. Additionally, significant differences were found in the TP of the sediment between these two habitats (*p* < 0.05, Figure 2). Furthermore, the average TP of the sediment in these two habitats exhibited a range from 0.83 g/kg (L01) to 2.39 g/kg (L07) and 0.50 g/kg (R04) to 1.18 g/kg (R01), respectively. These low concentrations showed the poor status of the sediment nutrient in these two habitats.

### 3.2. Bacterial α- and β-Diversity

An average of 95,603 reads were obtained from each sampling site. After trimming, screening, and removing chimeras, 83,647 high-quality sequences were obtained and then assigned to 2604 and 2910 OTUs for the lake and river habitats, respectively. A total of 2573 OTUs were shared by both communities. Good’s coverage was 96.93–99.30%, suggesting that the sequencing effort was sufficient to capture bacterial diversity. This was supported by rarefaction curves that approached an asymptote (Figure S1).

**Figure 3:**
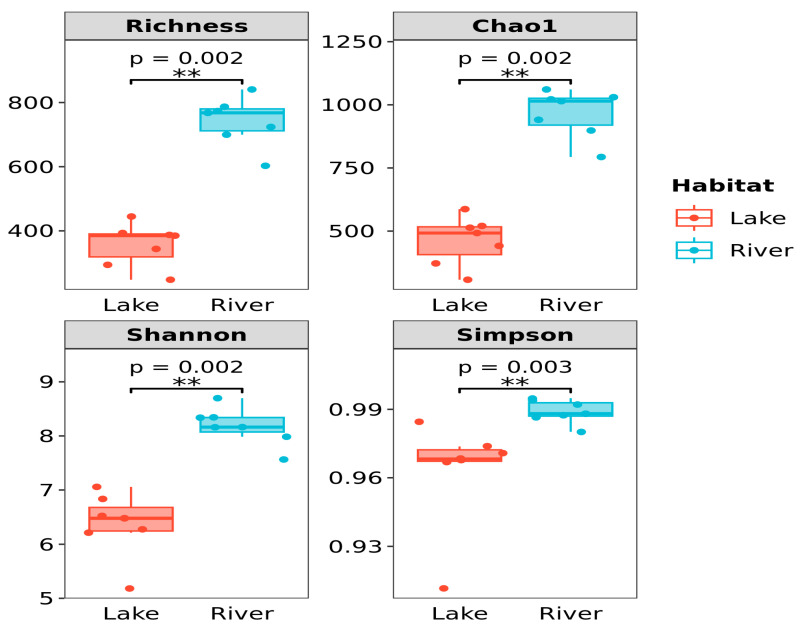
Comparisons of the bacterial α-diversity of the sediment between the lake and river habitats. **, *p* < 0.01.

**Figure 4:**
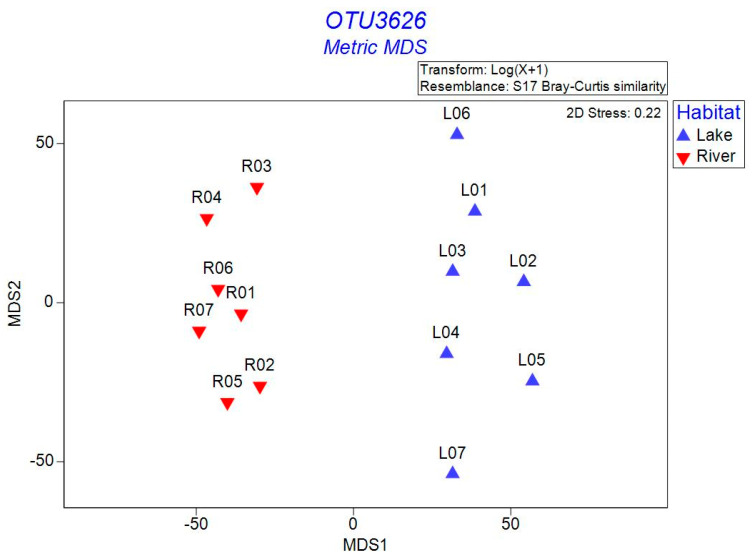
Comparisons of the bacterial β-diversity of the sediment between the lake and river habitats.

The bacterial α-diversity, including Richness, Chao1, Shannon, and Simpson indexes, of the sediment in the lake and river habitats were summarized in Figure 3. These values were significantly higher in the river habitats than in the lake habitats (*p* < 0.001). Additionally, MMDS was used to analyze the β-diversity patterns of bacterial communities in the sediment (Figure 4). Lake habitats were plotted on the right, whereas river habitats were on the left, suggesting that the bacterial communities in these two habitats were well separated. This result was also supported by the clustering analysis (Figure S2). The PERMANOVA test provided a similar result showing that the bacterial community structure was significantly different between these two habitats (*p* < 0.01). Notably, the volcanic lakes were more dispersed than the surrounding rivers, indicating greater variations in the sediment bacterial communities of the volcanic lakes.

### 3.3. Bacterial Taxonomy and Community Structure

**Figure 5:**
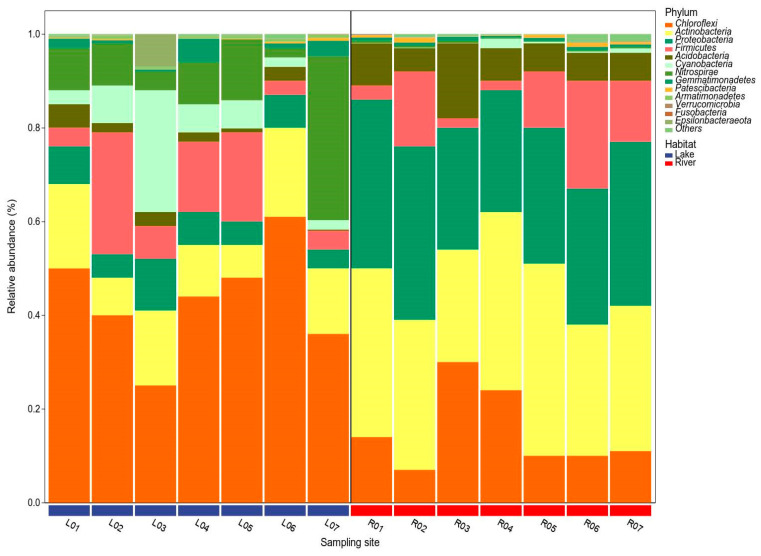
The relative abundance of sediment bacterial communities at the phylum level in the lake and river habitats. Only the top 13 phyla are shown at each sampling site, and the rest are defined as ‘Others’.

At the phylum level, the sediment of the lake and river habitats exhibited distinct BCC patterns (Figure 5). Within the lake habitats, sediment BCC was dominated by Chloroflexi (43.43%, average relative abundance), Actinobacteria (13.29%), Nitrospirae (11.58%), Firmicutes (11.15%), Cyanobacteria (7.57%), Proteobacteria (6.71%), and Acidobacteria (2.30%). In contrast, in the river habitats, sediment BCC was dominated by Actinobacteria (32.86%), Proteobacteria (31.14%), Chloroflexi (15.14%), Firmicutes (10.14%), and Acidobacteria (7.86%).

**Figure 6:**
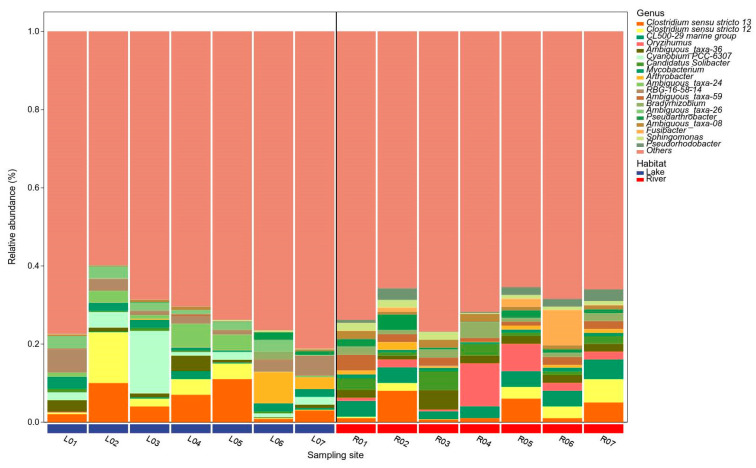
The relative abundance of sediment bacterial communities at the genus level in the lake and river habitats. At the genus level, only the top 19 dominants are exhibited, and the rest are defined as ‘Others’.

At the genus level, the sediment of the lake and river habitats also exhibited distinct BCC patterns (Figure 6). Within the lake habitats, *Clostridium sensu stricto 13* was the most abundant group, accounting for 5.40%, followed by *Cyanobium PCC-6307* (3.99%) and *Clostridium sensu stricto 12* (3.39%). In contrast, in the river habitats, *CL500-29 marine group* was the most abundant group, accounting for 3.71%, followed by *Oryzihumus* (3.61%), *Clostridium sensu stricto 13* (3.23%), and *Clostridium sensu stricto 12* (2.07%).

The cluster analysis showed a distinct separation of the sediment BCC from both the lake and river habitats (Figure S2). Both PERMANOVA analysis and ANOSIM showed that the differences in the sediment BCC between these two habitats were all statistically significant (*p* < 0.01).

### 3.4. Co-Occurrence Networks of Sediment Bacterial Communities

**Figure 7:**
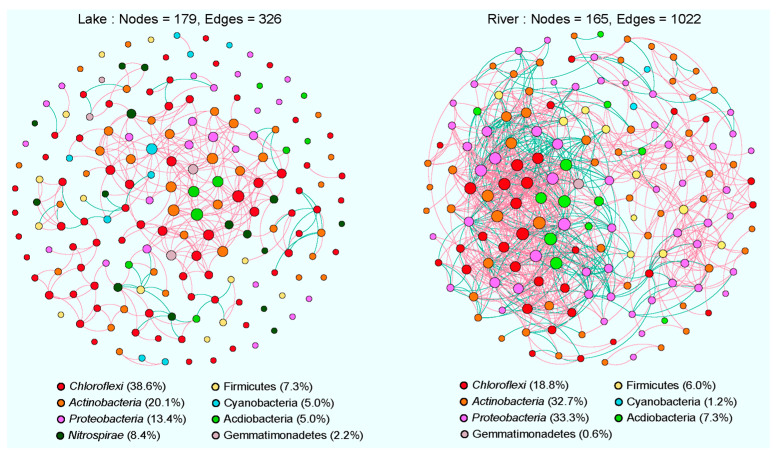
Co-occurrence networks of the sediment bacterial communities in the lake and river habitats. A connection represents a significant abs(r) > 0.85 (*p* < 0.01). The size of each node is proportional to the number of connections (i.e., degrees), and the nodes are colored according to different phyla. Red and green edges indicate positive and negative correlations, respectively.

The co-occurrence networks of sediment bacterial communities in the lake and river habitats were constructed separately to reveal the ecological interactions among different bacterial species (Figure 7). Based on correlation analysis, 326 edges with 179 nodes and 1022 edges with 165 nodes were found in the networks of lake and river bacterial communities, respectively (Table S2). The higher values of avgCC and modularity in lake and river networks than those in their related random networks imply that our constructed networks have “small-world” properties and a modular structure. It was confirmed by the value of the small-world coefficients (σ) of the lake (1.4) and river (3.2) networks with σ > 1 showing “small-world” properties [22].

The river network maintained higher values of AD and GD than the lake network (Table S2), suggesting more connections and interactions among the bacterial species of the sediment in the river network. Furthermore, the co-occurrence patterns of these two habitats were mostly positively structured (lake: 80.7%, river: 97.6%). The robustness analysis showed that the river (0.36 ± 0.02) network had a significantly higher (*p* < 0.05) robustness than the lake (0.32 ± 0.04) network, which suggested that the river network was more stable (Figure 7 and Table S2).

The majority of nodes in these two networks belonged to seven dominant phyla as follows: Chloroflexi, Actinobacteria, Proteobacteria, Nitrospirae, Firmicutes, Cyanobacteria, and Acidobacteria (Figure 7). Among them, Proteobacteria (33.3%) was the most prevalent phylum in the river network, whereas Chloroflexi (38.6%) was the most dominant phylum in the lake network. Keystone taxa were identified in these two networks by nodes with a high degree value and a low degree of betweenness centrality [23]. In the lake network, two OTUs belonging to the *Cyanobium PCC-6307* (Cyanobacteria) and the uncultured *Anaerolineaceae* (Chloroflexi) were identified as keystone species, while, in the river network, five OTUs belonging to the uncultured *Rhizobiaceae*, uncultured *Xanthobacteraceae*, *Devosia*, *Rhodobacter*, four of which belong to the Betaproteobacteria, and the *Pseudarthrobacter* of Actinobacteria, were identified as keystone species (Table S3). 

### 3.5. Environmental Drivers on Sediment Bacterial Communities

**Figure 8:**
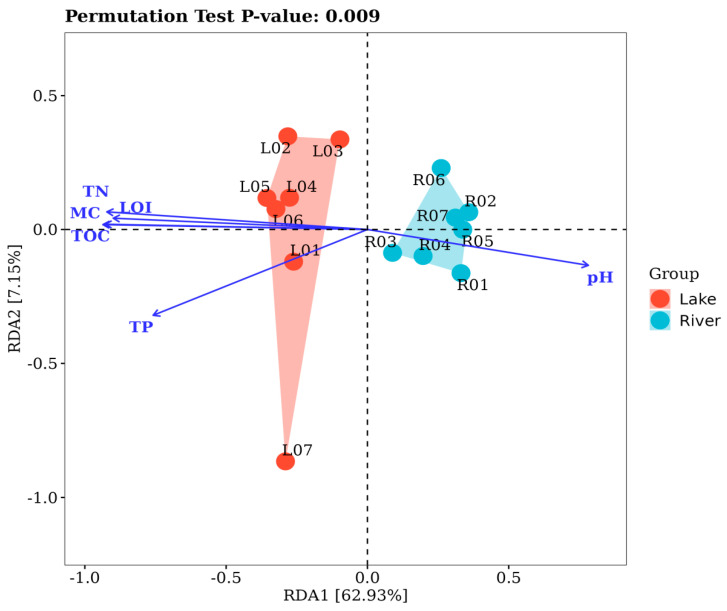
The RDA ordination showing the sediment BCC of the lake and river habitats in relation to significant environmental factors (*p* < 0.05). The sampling sites are shown as circulars. TN, total nitrogen; TP, total phosphorus; LOI, loss on ignition; MC, moisture content; TOC, total organic carbon.

We performed an RDA biplot analysis of the sediment BCC and the six environmental variables (pH, TN, TP, LOI, MC, and TOC) for lake and river habitats (Figure 8). The RDA biplot revealed that the pH, TN, TP, LOI, MC, and TOC were the important environmental factors affecting the variation of the sediment BCC between these two habitats. Among them, the TP was a significant environmental factor (*p* < 0.05), and the pH, TN, LOI, MC, and TOC were the highly significant environmental factors (*p* < 0.001) in shaping the variation of the sediment BCC. The eigenvalues of the first and second axes were 0.0527 and 0.0059, respectively, and these two axes explained 70.08% of the variation in the BCC of the sediment between these two habitats.

## 4. Discussion

### 4.1. Comparison of Sediment Bacterial Communities between Lake and River Habitats

In this study, we found that the bacterial α-diversity of the sediment in the river habitats were significantly higher than that of the lake habitats (Figure 3). This reason may be related to the high richness of the river habitats. Previous studies have suggested that the high richness observed in the river habitats may have resulted from the enormous movement of terrestrial, sediment, and periphyton microbiomes [24,25,26]. It is well known that terrestrial soil frequently contains diverse microbes that act as a regional microbial pool for its associated aquatic ecosystems [17]. Furthermore, the high environmental and spatial heterogeneity among different locations of rivers could attribute varied niches for diverse microbes living. Environmental variability includes the pH (6.61–7.38), TP (0.064–1.180 mg/g), and TOC (0.78–4.47 mg/g) and was hardly found in the lake habitats (Figure 1). Previous studies have reported that network modules (or clusters) might be understood as overlapping niches in which groups of species are more tightly coupled than others [27,28,29]. In this study, network topology analysis showed that the modularity of the river network was higher than that of the lake network (Table S2), implying more ecological niches for various microbes to perch in the river habitats.

Although the bacterial community structure of these two habitats differed significantly, we found a larger overlap of dominant groups in the sediment bacterial communities (Figure 5). For example, *Chloroflexi*, *Actinobacteria*, and *Proteobacteria* predominated in the sediment of both of these two habitats. These phylogenetic taxa also dominated in the sediment of other aquatic habitats in China, North America, and Europe [6,30,31,32,33,34,35]. These findings indicate the presence of a permanent and widespread dominant sub-community in these two habitats. At the genus level, *Clostridium sensu stricto 13* and *Clostridium sensu stricto 12* were found as the prominent phylotypes. Their prominence could be related to the presence of *Cyanobacteria* in the sediment of the lake and river habitats (Figure 6). Previous studies have also found that *Clostridium sensu stricto* might play important roles in the decomposition of algal biomass, was positively linked with the DOC and turbidity, and was negatively correlated with pH and DO [36]. This is consistent with the higher DOC and lower pH of the sediment in these two habitats in our study. However, we also found the genus *Cyanobium PCC-6307* affiliated with the phylum *Cyanobacteria* abundantly present in the sediment of the lake habitats. This reason might be mainly due to the content of TN, TP, and TOC in the sediment of the lake habitats being significantly higher than that of the river habitats (*p* < 0.01, Figure 2). Previous studies have indicated that as the concentration of nutrients such as nitrogen and phosphorus increases, the biomass of benthic sediment algae will significantly increase [37,38,39]. 

### 4.2. Comparison of Co-Occurrence Networks between Lake and River Habitats 

Network analysis improves the knowledge of microorganism interactions and predicts the aggregation condition of microorganisms in environments [40]. In the current study, the co-occurrence networks in these two habitats were mainly composed of positive correlations. This positive correlative trend has been observed on numerous occasions in the bacterial co-occurrence networks from various habitats [41,42]. Previous research has demonstrated that this positive relationship can be interpreted as cross-feeding, co-colonization, and co-aggregation [28] and can, in turn, be correlated with phylogeny [43]. 

The keystone taxa are of great importance in maintaining the stability of microbial ecosystems [40,44]. In the lake network, the keystone genera were *Cyanobium PCC-6307* and *Uncultured Anaerolineaceae*. *Cyanobium PCC-6307* appears to be important in the amino acid metabolism [45]. As the most common keystone genus in the river network, *Devosia* has been identified as a bacterial species capable of establishing a nitrogen-fixing root nodule symbiosis with the aquatic legume N. natans (L.f.) Druce [46]. *Pedobacter* has been proposed to play a crucial role in functioning as an energy-converting ferredoxin [47]. *Pseudarthrobacter* has several abilities, including organic matter degradation, nitrogen fixation, and biological defense [48]. In our study, the number of nodes (179) and edges (326) in the lake network were less than in the river network (165 nodes and 1022 edges), indicating that the river network was more complex and coherent than the lake network. 

Additionally, the river network was more fragmented (modularity: 0.695) than the lake network (modularity: 0.458). The variations in the co-occurrence networks between these two habitats may be driven by the mechanisms related to metacommunity dynamics and biodiversity [49,50,51]. According to the notion of metacommunity [52], bacterial communities in the lotic ecosystem are partially driven by a common terrestrial origin of aquatic communities [24]. Therefore, the river habitats have a larger metacommunity size than the lake habitats due to their limited contributing area. Besemer et al. [53] also revealed that these impacts may increase the biodiversity in rivers, which is demonstrated by the current findings (Figure 3). Higher biodiversity, in turn, promotes interactions in bacterial communities [54] and increases their co-occurrence pattern (lower fragmentation) [55]. Thus, the lake was more highly fragmented than the river in the present study.

### 4.3. Environmental Factors in Relation to Sediment Microbial Communities

The RDA results indicate that both physical [pH and MC] and nutrition-related factors (TN, TP, LOI, and TOC) had a significant effect on the variation of the sediment bacterial communities between these two habitats (Figure 8), implying that local environmental factors play a key role in structuring the sediment bacterial communities. Previous studies of many sizes, spanning from local to regional and global levels, have consistently documented the effect of environmental conditions, including pH and nutrient loadings, on the bacterial communities of the sediment [56,57,58,59,60,61,62,63,64]. Interestingly, in this study, we observed that five environmental parameters (TN, TP, LOI, MC, and TOC) of the sediment in the lake habitats were all significantly higher than those of the river habitats (*p* < 0.01, Figure 2). The high contents of nutrients in the lake habitats could be attributed to anthropogenic activities for the lakes of Arxan, such as increasing tourism, fish-farming, and runoff from livestock excrement such as cattle and sheep [5]. Previous studies have reported that increasing anthropogenic activity can lead to a decline in bacterial diversity based on the assessment of over 200 lakes [65]. This is in agreement with the findings of our study that the lower bacterial α-diversity of the sediment was observed in the lake habitats.

## Data Availability

All raw sequence data obtained in this study have been deposited at the Sequence Read Archive database of the National Center for Biotechnology Information under accession number PRJNA1032410. These data are publicly accessible at http://www.ncbi.nlm.nih.gov/.

## Supplementary Materials

The following supporting information can be downloaded at: https://www.mdpi.com/article/10.3390/microorganisms12071435/s1, Figure S1: Rarefaction curves for the 14 different sediment samples analyzed using the Chao1 bias-corrected estimator; Figure S2: UPGMA result based on the unweighted Unifrac metric at the phylum level. The hierarchical clustering structure helps to determine the similarity of the sediment bacterial communities between these two habitats; Table S1: The longitude and latitude of sampling sites in the Arxan UNESCO Global Geopark; Table S2: Major topological attributes of co-occurrence network within the sediment BCC in lake and river habitats; Table S3: List of keystone nodes and their taxonomic information in the lake and river network.
