# Supplementary material for: Comparing Sediment Bacterial Communities of Volcanic Lakes and Surrounding Rivers in Inner Mongolia Autonomous Region, Northeastern China
Source: Microorganisms. 2024 Jul 15;12(7):1435. doi: 10.3390/microorganisms12071435 (PMC11278812; doi:10.3390/microorganisms12071435)

Table S1 The longitude and latitude of sampling sites in the Arxan UNESCO Global Geopark.

Samples	Longitude	Latitude
L01	120°40′16″	47°24′6″
L02	120°29′56″	47°23′37″
L03	120°33′53″	47°24′53″
L04	120°26′49″	47°20′39″
L05	120°20′9″	47°17′38″
L06	120°24′22″	47°18′58″
L07	120°38′46″	47°27′23″
R01	119°45′40″	47°18′16″
R02	119°40′17″	47°24′17″
R03	119°41′11″	47°24′36″
R04	119°52′53″	47°18′24″
R05	119°58′7″	47°19′37″
R06	119°55′16″	47°19′28″
R07	120°12′4″	47°20′38″

Table S2 Major topological attributes of co-occurrence network within the sediment BCC in lake and river habitats.

	Empirical network											Random network		
	N	E		Modularity	avgCC	APL	ND	GD	AD	σ	Robustness	Modularity	avgCC	APL
		Positive	Negative											
Lake	179	263 (80.7%)	63 (19.3%)	0.458	0.584	2.18	8	0.418	15.105	1.5	0.32 ± 0.04	0.208	0.365	2.02
River	165	997 (97.6%)	25 (2.4%)	0.695	0.375	5.74	14	0.035	3.482	2.4	0.36 ± 0.02	0.432	0.055	2.36

N: No. of nodes; E: No. of edges; avgCC: Average clustering coefficient; APL: Average path length; ND: network diameter; AD: Average degree; GD: Graph density;

σ: small-word coefficient, σ = (avgCC/avgCCr)/(APL/APLr) and σ > 1 indicates "small-world" properties, i.e., high interconnectivity and high efficiency. Subscript r indicates the properties of the random network.

Table S3 List of keystone nodes and their taxonomic information in the lake and river network.

Nodes	network	Phylum	Class	Order	Family	Genus
OTU0904	Lake	*Cyanobacteria*	*Oxyphotobacteria*	*Synechococcales*	*Cyanobiaceae*	*Cyanobium PCC-6307*
OTU0607	Lake	*Chloroflexi*	*Anaerolineae*	*Anaerolineales*	*Anaerolineaceae*	*Uncultured Anaerolineaceae*
OTU0698	River	*Proteobacteria*	*Alphaproteobacteria*	*Rhizobiales*	*Rhizobiaceae*	*uncultured*
OTU0798	River	*Proteobacteria*	*Alphaproteobacteria*	*Rhizobiales*	*Devosiaceae*	*Devosia*
OTU0817	River	*Proteobacteria*	*Alphaproteobacteria*	*Rhizobiales*	*Xanthobacteraceae*	*uncultured*
OTU1178	River	*Proteobacteria*	*Alphaproteobacteria*	*Rhodobacterales*	*Rhodobacteraceae*	*Rhodobacter*
OTU1233	River	*Actinobacteria*	*Actinobacteria*	*Micrococcales*	*Micrococcaceae*	*Pseudarthrobacter*

Figure legends

Figure S1. Rarefaction curves for the 14 different sediment samples analyzed using the Chao1 bias-corrected estimator.

Figure S2. UPGMA result based on the unweighted Unifrac metric at the phylum level. The hierarchical clustering structure helps to determine the similarity of the sediment bacterial communities between these two habitats.

Figure S1.

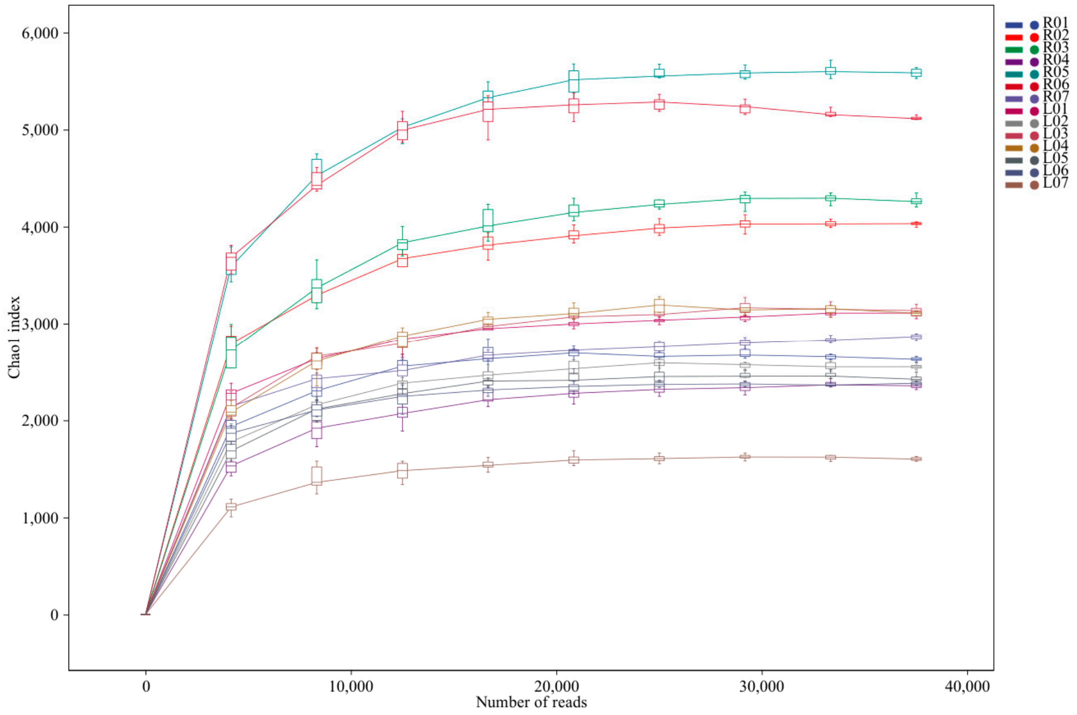

Figure S2.

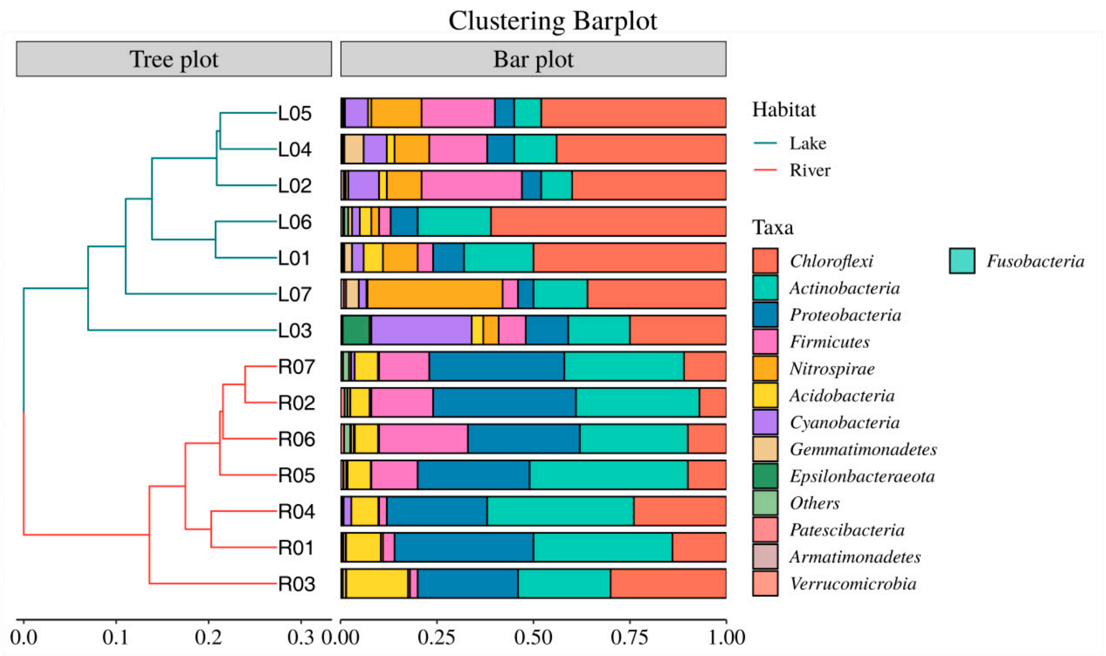

Supplement: Supplementary file 1 [file microorganisms-12-01435-s001.zip › microorganisms-3094358-supplementary.pdf]
